# Heterotopic mineral deposits in intact rat Achilles tendons are characterized by a unique fiber-like structure

**DOI:** 10.1016/j.yjsbx.2023.100087

**Published:** 2023-02-24

**Authors:** Maria Pierantoni, Malin Hammerman, Isabella Silva Barreto, Linnea Andersson, Vladimir Novak, Hanna Isaksson, Pernilla Eliasson

**Affiliations:** a Department of Biomedical Engineering, Lund University, Box 118, 221 00 Lund, Sweden; b Department of Biomedical and Clinical Sciences, Linköping University, 581 83 Linköping, Sweden; c Swiss Light Source, Paul Scherrer Institute, CH-5232 Villigen, Switzerland; d Department of Orthopaedics, Sahlgrenska University Hospital, Gothenburg, Sweden

**Keywords:** Pathological mineralization, Calcification, Collagen, Phase contrast enhanced synchrotron X-ray tomography, Fibers, HO, Heterotopic Ossification

## Abstract

Heterotopic mineralization entails pathological mineral formation inside soft tissues. In human tendons mineralization is often associated with tendinopathies, tendon weakness and pain. In Achilles tendons, mineralization is considered to occur through heterotopic ossification (HO) primarily in response to tendon pathologies. However, refined details regarding HO deposition and microstructure are unknown. In this study, we characterize HO in intact rat Achilles tendons through high-resolution phase contrast enhanced synchrotron X-ray tomography. Furthermore, we test the potential of studying local tissue injury by needling intact Achilles tendons and the relation between tissue microdamage and HO. The results show that HO occurs in all intact Achilles tendons at 16 weeks of age. HO deposits are characterized by an elongated ellipsoidal shape and by a fiber-like internal structure which suggests that some collagen fibers have mineralized. The data indicates that deposition along fibers initiates in the pericellular area, and propagates into the intercellular area. Within HO deposits cells are larger and more rounded compared to tenocytes between unmineralized fibers, which are fewer and elongated. The results also indicate that multiple HO deposits may merge into bigger structures with time by accession along unmineralized fibers. Furthermore, the presence of unmineralized regions within the deposits may indicate that HOs are not only growing, but mineral resorption may also occur. Additionally, phase contrast synchrotron X-ray tomography allowed to distinguish microdamage at the fiber level in response to needling. The needle injury protocol could in the future enable to elucidate the relation between local inflammation, microdamage, and HO deposition.

## Highlights

• The collagen fiber structure of the Achilles tendon is preserved within HO deposits during calcification.
• Phase contrast enhanced synchrotron X-ray tomography enables to directly visualize microdamage at the collagen fiber level and early formation of HO deposits.
• In Achilles tendons, pathological mineralization initiates in the pericellular area and propagates into the intercellular area.
• Multiple HO deposits appear to merge by accession along unmineralized fibers.
• The proposed needling protocol will enable to elucidate the relation between local inflammation, microdamage and pathological mineralization.

## Graphical abstract

**Figure:**
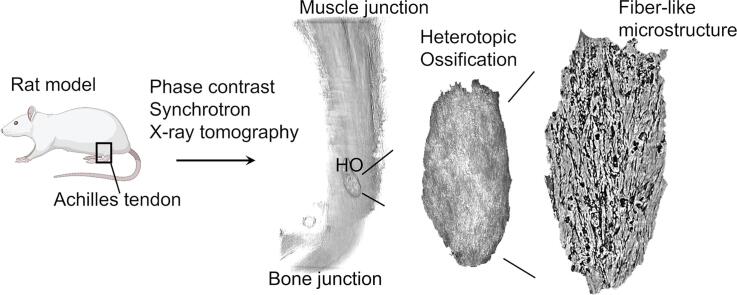

## 1 Introduction

Tendinopathies are common musculoskeletal pathologies (Snedeker and Foolen, 2017, Sharma and Maffulli, 2005). Heterotopic mineralization in intact tendons is one possible feature associated with tendinopathies that in some cases give rise to pain and tendon weakness (Järvinen et al., 1997, O’Brien et al., 2012). Heterotopic mineralization can be the result of calcification or ossification, where calcification is characterized by deposition of calcium salts, whereas ossification occurs when bone-like deposits form (Chan et al., 2002). Calcification and ossification cannot be easily distinguished by medical radiography and the two types of mineralization may occur simultaneously (Strumia et al., 1997).

Previous studies have shown that mineralization in tendons is most likely occurring through Heterotopic Ossification (HO) (O’Brien et al., 2012, Lin et al., 2010, Fenwick et al., 2002, Lui et al., 2009, Lui et al., 2009, Xu et al., 2022). In humans Achilles tendons, HO is more often observed after a severe trauma, but this could be due to lack of systematic screening in absence of trauma. Recently, the presence of HO was reported after surgically repaired Achilles tendon ruptures in almost 20% of the patients (Magnusson et al., 2020). We still do not fully understand the underlying mechanisms of HO in tendons, and there are no specific treatments to limit HO deposition, aside from surgical removal. The specific biological mechanisms guiding deposition of HO need to be further investigated (Xu et al., 2022). Recent studies indicate that inflammation could alter the microenvironment and contribute to HO (Sung Hsieh et al., 2017) and that accumulation of inflammation-associated cells, e.g. macrophages, can promote HO deposition (Convente et al., 2018, Li et al., 2021). HO is currently believed to occur through intramembranous ossification or endochondral ossification (Karsenty and Wagner, 2002). Intramembranous ossification occurs when mesenchymal cells condense and differentiate into osteoblasts, whereas during endochondral ossification mesenchymal stem cells differentiate into chondrocytes forming cartilage which serves as a template and guides the mineralisation process. In Achilles tendons, both endochondral ossification and intramembranous ossification have been reported (Richards et al., 2008). However, details regarding HO etiology, morphology and the process of deposition are still unknown.

Animal models are fundamental for closing some of our knowledge gaps with regards to the formation of HO deposits in tendons, specifically rat and mouse models (Lin et al., 2010, Hammerman et al., 2018, Khayyeri et al., 2017, Khayyeri et al., 2020, Hannallah et al., 2004). Several animal studies have indicated that HO deposition during tendon healing is dictated by endochondral ossification (Lin et al., 2010, Lui et al., 2009, Rooney et al., 1992, Asai et al., 2014, Suzuki et al., 2016, Zhang et al., 2020). However, very little is known about the occurrence of HO in intact tendons, and the mechanisms guiding the formation of HO deposits in the absence of tendon rupture remain largely unknown. We use a rat model in combination with cutting-edge high-resolution phase contrast enhanced synchrotron X-ray tomography to increase our knowledge of the process of HO deposition at the microscale in intact Achilles tendons. This technique offers the unique possibility to rapidly image relatively large volumes with micrometer resolution, without the need of invasive sample preparations (Gonzalez-Tendero et al., 2017, Horng et al., 2014, Zhou et al., 2018, Disney et al., 2017, Eckermann et al., 2020, Gradl et al., 2019, Horng et al., 2021). Because of low absorption and small density differences, imaging soft biological materials by conventional tomography is challenging. However, in phase contrast enhanced synchrotron X-ray tomography the contrast is amplified by the wave propagation phenomena (Morgan et al., 2010, Cloetens et al., 1999). Thus, we use this technique to visualize in 3D at high resolution tendon soft tissue and to capture very early stages of HO, when very little mineral is yet deposited. The aim of this study is to present clear evidence that HO occur in intact Achilles tendons and to characterize 3D microstructure of the deposits in relation to the soft unmineralized collagen fibers. The results provide a better understanding of the microstructure and mineral deposition pattern. Furthermore, since tissue damage possibly contributes to HO deposition (Sung Hsieh et al., 2017, O’Brien et al., 2013), we investigate the feasibility of using a needle injury protocol in rat Achilles tendons in combination with high-resolution phase contrast enhanced synchrotron X-ray tomography to explore the possibility to study HO formation, microdamage, and early stages of mineral deposition.

## 2 Methods

### 2.1 Animal model

Female Sprague-Dawley rats, 12 weeks old, (n = 16) were used (Janvier, Le Genest-Saint-Isle, France). The rats were kept in pairs, with a light–dark cycle of 12 h and controlled humidity (55%) and temperature (22 °C). Food was provided *ad libitum*. Two separate sets of experiments were performed: 1) characterization of HO deposits in intact Achilles tendons (n = 7) where no intervention was performed on the tendons (experiment 1 in table 1), and 2) a needle injury protocol (n = 9) to investigate if we could detect the traces of microdamage and its relation to deposition of HO in tendons (experiment 2 in table 1). A similar needle injury was previously performed on rat healing tendons (Hammerman et al., 2014, Hammerman et al., 2015) and it was used in mice to study HO (O’Brien et al., 2012, O’Brien et al., 2013). For the needle injury experiment the rats were divided in 3 groups: a control group (no needling, n = 3), needled 5 times (n = 3) and needled 20 times (n = 3). Animals were anesthetized with isoflurane and did not receive any analgesics before or after the needling. Needle injury was induced by percutaneous penetrations into the mid-portion of the right Achilles tendon using an insulin needle (0.25 mm in diameter, size 31G), followed by 5 needle movements inside the tendon tissue in different directions. For the 5 times group, needling was performed through the skin from the lateral side, while the 20 times group was performed through the lateral, medial, proximal, and distal side resulting in a total of 20 punctures into the tendon. Additionally, animals from a third experiment (intact, no needling) were used for histological evaluation. After four weeks the rats were euthanized and the tendons along with the muscle complex and the calcaneal bone were dissected, placed in a phosphate buffered saline solution (PBS), and frozen at −20 °C. The study was approved by the Regional Ethics Committee for animal experiments in Linköping, Sweden (Jordbruksverket, ID1424 and ID6012).

### 2.2 Phase contrast enhanced synchrotron X-ray tomography (SR-PhC-μCT)

The tendons from experiment 1 and 2 were thawed and mounted in 2 ml Eppendorf tubes filled with PBS, following previous studies (Pierantoni et al., 2021). The imaging was performed at the X02DA TOMCAT beamline at the Swiss Light Source (SLS), Paul Scherrer Institute (Villigen, Switzerland) (Stampanoni et al., 2006). The images were acquired using a High Numerical Aperture Microscope setup (4x magnification, field of view of 4.2 mm × 3.5 mm and final pixel size of 1.63 µm). The optical setup was coupled to a LuAG:Ce scintillator screen of 150 μm. A propagation distance of 150 mm was chosen, the X-ray energy was set at 15 keV, 2001 projections were acquired over 180° of continuous rotation, and the exposure time was 33 ms. The samples were imaged in two–three consecutive locations, starting from the bone junction and moving up to the muscle junction of the tendon. Projections were collected by a pco.edge 5.5 Camera and corrected with dark and flat-field images. The projected density of the sample was calculated using the Paganin phase retrieval for homogeneous objects (Paganin et al., 2002). The tomographic reconstruction was performed using a Fourier based regridding algorithm (Marone and Stampanoni, 2012).

### 2.3 SR-PhC-μCT image processing

To visualize the whole tendons, the volumes were stitched together using the BigStitcher software package for ImageJ (Hörl et al., 2019). Then, volume renderings were performed in Dragonfly (v 4.6, ORS software) (Dragonfly 4.6 ORS). The quantitative image analysis was performed in MATLAB. HO deposits and tendon tissue were segmented by binarization. The threshold values were adjusted for each type of tissue and morphological operations of opening, closing and gap filling were performed iteratively to optimize the segmentation output. The volumes of HO deposit and total tendon volumes were then calculated by summing the white pixels in each binary segmented image. The volume fraction of HO deposits was defined as the percentage of tendon tissue occupied by HO deposits and was calculated as the total volume of HO deposits divided by the total tendon volume. The location of the deposits was calculated as the distance from the HO deposit center to the calcaneal bone. To compare the HO deposit locations between different tendons the distance was normalized to the total tendon length so that the end of the calcaneal bone was equal to 0 and the start of the muscle complex to 1.

### 2.4 Histology

Tendons from experiment 3, which were also part of another study (Khayyeri et al., 2017), were fixed in 4% formaldehyde for 48 h, followed by a standard protocol with ethanol dehydration and paraffin embedding. Embedded tendons were sectioned longitudinally to 3 μm thick slices and stained with Alcian blue (pH 2,5), for visualization of proteoglycans, and Picrosirius red, for visualization of collagen fiber structure (Khayyeri et al., 2017). The stained specimens were examined using an Olympus BX43 light microscope.

## 3 Results

### 3.1 HO in intact tendons

**Fig. 1:**
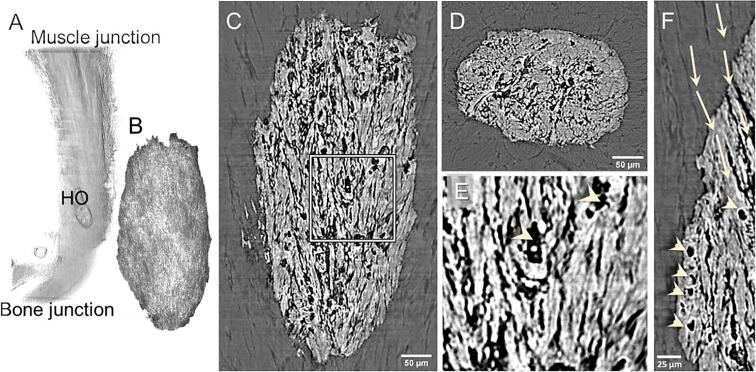
**HO deposits in intact Achilles tendons are characterized by a fiber-like structure.** A) Volume rendering showing the location of the HO deposit in the whole tendon (from bone to muscle junction). B) Volume rendering of one deposit of HO. Longitudinal (C) and cross-section (D) slices showing the internal fiber-like structure of the HO deposit. E) magnification of the area in the square in C) and F) magnification showing the transition from collagen fibers to mineralized fibers in the tendon tissue. Arrowheads indicate cell-like lacunae within the deposit and the arrows show how the fiber structure is maintained moving from the soft collagen tissue into the mineralized deposit.

HO deposits were present in all intact tendons on the anterior side of the distal third of the tendon (table 1and Fig. 1A). All tendons contained between 1 and 6 individual HO deposits with an average size of 0.03 ± 0.02 mm^3^, occupying about 1% of the tendon total volume (Table 1).

**Table 1:** **Quantification of HO deposit volumes, volume fractions, and HO locations.** All data is presented as mean ± SD. For determining HO deposit location, the tendon length was normalized so that 0 is the bone junction and 1 is the muscle junction.

Experiment	Samples	Tendon length (cm)	Number of deposits (tendons)	Volume of individual deposits (mm^3^)	Total deposit volume (mm^3^)	Volume fraction (%)	HO location (cm)
1	Intact tendons (n = 7)	1.20 ± 0.08	1(3), 2(2), 3(1), 6(1)	0.03 ± 0.02	0.08 ± 0.04	0.9 ± 0.6 %	0.29 ± 0.13

2	Control (n = 3)	1.21 ± 0.17	1(2), 2(1)	0.03 ± 0.03	0.04 ± 0.03	0.3 ± 0.3 %	0.31 ± 0.07
	Needling 5 times (n = 3)	1.21 ± 0.13	1 (2), 3(1)	0.07 ± 0.08	0.10 ± 0.09	0.7 ± 0.8 %	0.25 ± 0.09
	Needling 20 times (n = 3)	1.14 ± 0.11	1 (2), 2(1)	0.06 ± 0.04	0.10 ± 0.01	0.7 ± 0.2 %	0.30 ± 0.10

All HO deposits were characterized by an elongated ellipsoidal morphology and a fiber-like internal structure (Fig. 1 C-F, video 1). Some of the tendon collagen fibers had mineralized while preserving their fiber structure and could be tracked from the tendon soft tissue into the HO deposits (Fig. 1F arrows). Within the HO deposits, round unmineralized lacunae, of about 15 µm diameter, could be observed possibly indicating the presence of cells (Fig. 1 E, F arrowheads).

**Fig. 2:**
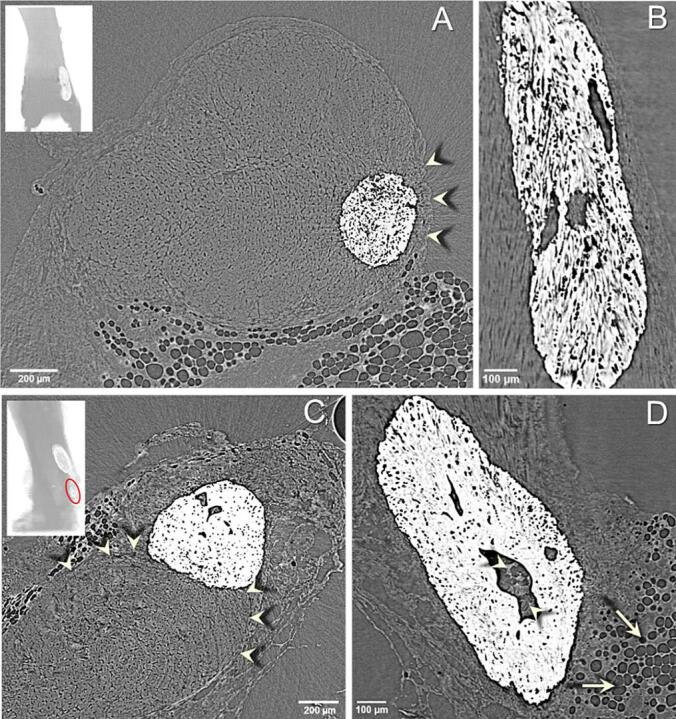
**HO deposits containing unmineralized gaps.** A) Tendon cross-section showing the location of the HO deposit within the tendon margins (arrowheads). B) Longitudinal view showing three gaps within one HO deposit. C) Tendon cross-section showing the HO deposit bulging out from the tendon margins (arrowheads) into the surrounding tissue. D) Gap within a deposit containing adipocyte-like structures (arrowheads) similar to adipose tissue found outside the tendon (arrows). Inserts: longitudinal views showing the position of the deposits from the calcaneus bone on A) the bottom anterior and C) lateral views, the circle indicates the deposit shown in the main panel.

Furthermore, in 11 out of 29 (∼40%) of the analyzed HO deposits large unmineralized gaps were observed within the calcified structure (Fig. 2). In most cases the deposits were located within the tendon margins (Fig. 2A) and unmineralized fibers were visible in the gaps (Fig. 2B). Additionally, adipocyte-like structures were found in some gaps, but only when the minerals bulged out from the tendons (Fig. 2C-D).

### 2.2 HO deposits in micro-injured tendons

**Fig. 3:**
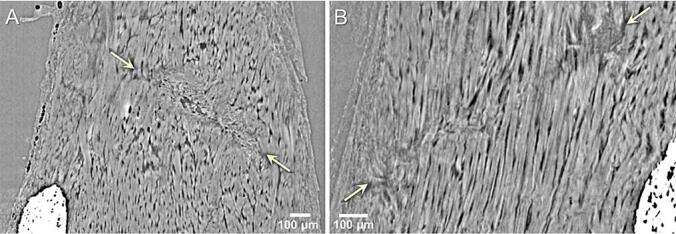
**Microdamage in the collagen structure induced by the needling was visible after 4 weeks.** A) 5 times needled tendon and B) 20 times needled tendon. The arrows indicate the beginning and end of the damaged region. In both tendons HO deposits in the proximity of the needled regions were observed.

Regions where the needle had penetrated the soft tendon tissue, could be identified as microdamage in the collagen fiber structure and HO deposits were found in the vicinity of the microdamage (Fig. 3).

**Fig. 4:**
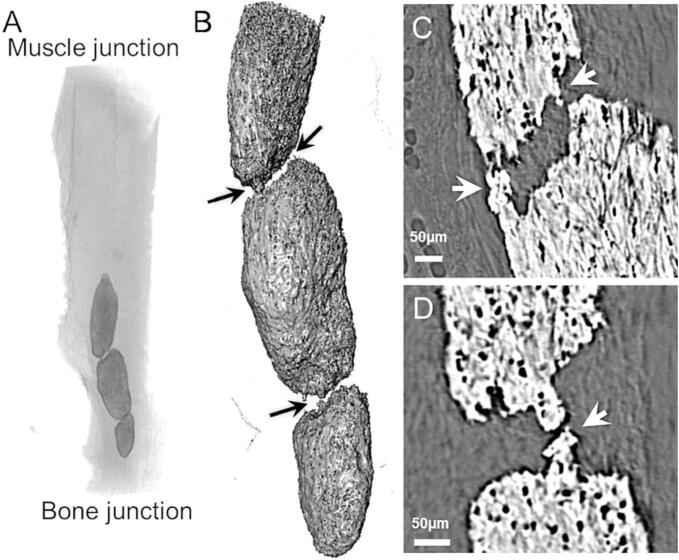
**Merging deposits of HO.** A) Volume rendering showing the location of three merging HO deposits in a 5 times needled tendon. B) Volume rendering magnification of the three merging deposits in A. C-D) Longitudinal magnifications showing the merging (arrows) between the top and middle deposits (C) and between the middle and bottom deposit (D).

HO deposits were present in all studied tendons and were located within the distal third of the tendon, with one exception where the deposits extended up to almost half of the tendon length (Fig. 4 and Table 1). As in the uninjured tendons, all HO deposits were characterized by a fiber-like internal structure. The sizes of the deposits were comparable for the two needled groups, occupying about 0.7% of the total volume in injured tendons, compared to about 0.3% in the controls (Table 1). However, the group size prevented further statistical analysis. One interesting aspect observed in two of the needled tendons (one 5 times and one 20 times) was that multiple HO deposits were merging. In areas between individual deposits, the mineralization seems to proceed along some of the fibers, creating points of contact (Fig. 4 B-D).

**Fig. 5:**
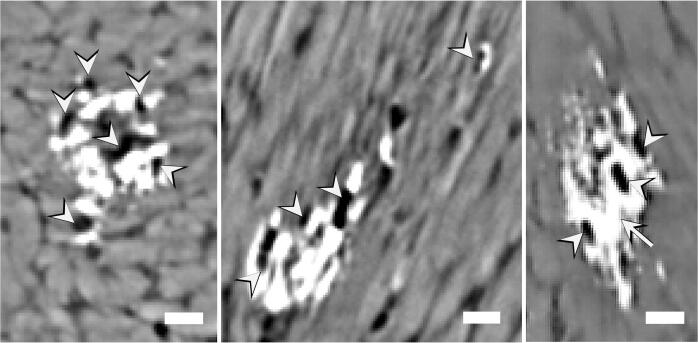
**Formation of an HO deposit in a tendon needled 5 times.** From left to right: cross-sectional, left and right views of the deposit showing that HO may start around cells (arrowheads) and propagates along the fibers in between them (arrow). Scale bars = 25 µm.

Moreover, early stages of HO could be identified in one of the tendons needled 5 times (Fig. 5, video 2). HO occurred at about half of the tendon lengths at the lateral edge of the tendon, close to where the needle was inserted. However, microdamage from needling was not visible in this tendon. Additionally, the data seem to indicate that mineral deposition starts around cells (Fig. 5 arrowhead) and propagates along fibers in between them (Fig. 5 arrow).

### 2.3 Histological characterization of HO deposits in intact tendons

**Fig. 6:**
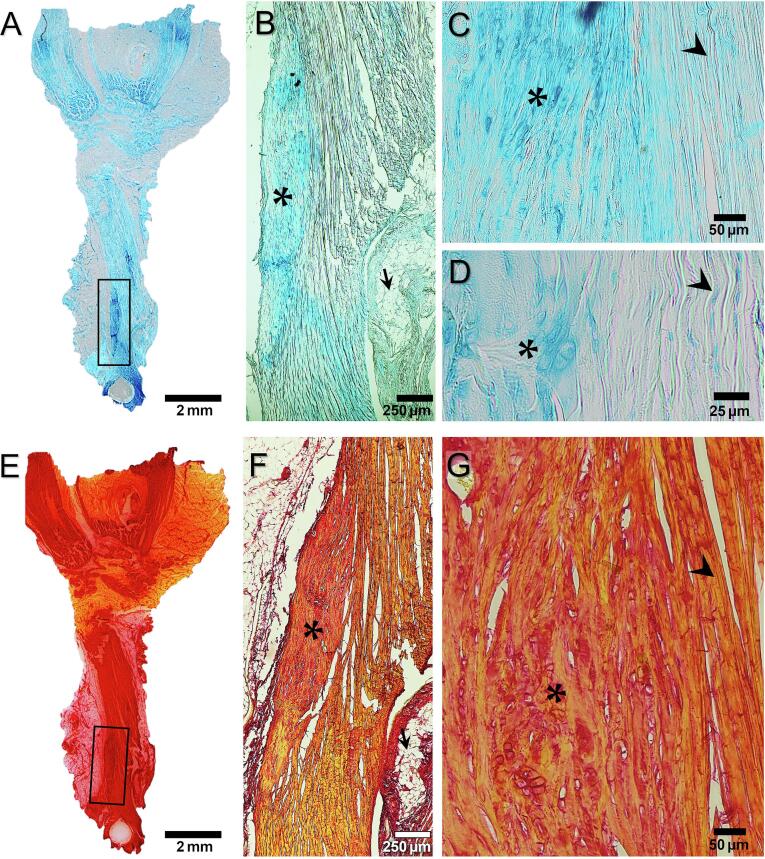
**Histology of HO deposits in intact tendons.** The tendons were stained with Alcian blue (A-D) or Picro Sirius Red (E-G). A and E) overview of the whole tendon, B and F) the HO deposit (*) was located at the distal side of the tendon close the surface. The mineralized region was denser and characterized by areas which were darker than the unmineralized fibers. The arrows indicate the presence of adipose tissue around the tendon. C) An increased number of cells is present in the HO (*) compared to the surrounding tissue (arrowhead). D) Within the mineralized region rounded cells (*) were observed. These cells were bigger than the elongated tenocytes (arrowhead) present in between unmineralized fibers. G) Within the HO deposit a fiber-like structure was preserved but the fibers are more disorganized and discontinuous (*) than the highly oriented unmineralized fibers (arrowhead). (For interpretation of the references to colour in this figure legend, the reader is referred to the web version of this article.)

HO appeared as darker blue areas in tendons stained with Alcian blue, indicating an increase of proteoglycans in these regions and thus a more cartilaginous tissue in the correspondence of the mineralized regions (Fig. 6A). Within the HO deposits an increased number of larger and rounded chondrocyte like cells was present compared to the fewer elongated tenocytes located between the tendon unmineralized collagen fibers (Fig. 6 B and C).

Picro Sirius Red staining showed that collagen fibers were preserved in the mineralized regions (Fig. 6 E-G). However, within the HO deposit, fibers were stained darker (red) than the unmineralized fibers which were more yellow (Fig. 6F). The fibers within HO deposit appeared more crimped and discontinuous (possibly in part due to the presence of numerous large cells) than in the adjacent unmineralized tissue, where fibers were highly aligned along the tendon main axes (Fig. 6G).

## 4 Discussion

In this study, the microstructure of HO deposits in intact rat Achilles tendons was characterized in detail. Deposits of HO were present in all tendons, had an elongated ellipsoidal shape, and occupied up to 1% of the tendon volume. They were mostly located on the anterior distal part close to the tendon-surface, in some cases protruding out from the tendon. The HO deposits were characterized by a fiber-like structure in which some fibers from the tendon soft collagen matrix had become mineralized. The structure of the deposits strongly resembles the one of calcified avian tendons where the calcification occur hierarchically along the collagen fibrils, fibers, and bundles (Fratzl et al., 2003, Landis and Song, 1991). Questions on how mineralization occurs in rat intact tendons and what factors trigger this process remain open. However, the fiber-like structure is unique for intact tendons and differs largely from the bone-like deposits observed this far in healing tendons after tendon rupture or transection (Lin et al., 2010, Zhang et al., 2020). This indicates that HO could proceed through different pathways in intact and injured tendons, but this needs further investigation.

In vitro studies have shown that tenocytes have a predisposition to differentiate into chondrocyte-like cells (Darrieutort-Laffite et al., 2019). In our study small ellipsoidal gaps were distinguished within the HO deposit structure (Fig. 5). These gaps (or cell lacunae) may indicate the presence of inflammatory cells, such as macrophages or masts, that can promote HO deposition (Convente et al., 2018). However, the lacunae could also consist of chondrocytes or tenocytes, as suggested by their locations, sizes, and organization (Pierantoni et al., 2021, Laudier et al., 2007). A recent study has shown that in naturally mineralizing turkey tendons, calcification occurs along fibrils in correspondence of canaliculi which are radiating from tenocytes (Zou et al., 2020). Our histological results show that cells within the mineralized regions are more rounded and bigger (possibly chondrocyte-like cells), and more densely organized compared to the tenocytes observed in the unmineralized tissue. Increased Alcain blue staining also suggests a more cartilaginous tissue. Additionally, HO could be the outcome of stem cell or progenitor cell differentiation from a group of tendon progenitor cells (Feng et al., 2020). Previous studies have also shown that BMP signaling could be triggering HO (Xu et al., 2022, Kaplan and Shore, 2000). Furthermore, both mechanical stimuli and inflammation could contribute to initiate mineral deposition by activating quiescent stem cells and the mTORC1 complex (Rodgers et al., 2014, Chen et al., 2017, Fu et al., 2022). However, further investigation is required to gain a full understanding of the complex process of HO. Additional studies may improve our understanding of pathological ossification mechanisms in vertebrate tissues by considering the possibility of the existence of similarities between pathological extracellular calcification along collagen fibrils/fibers in mammal intact tendons and physiological mineralization in avian tendons.

Large unmineralized areas were present within about 40% of the HO deposits. These unmineralized regions could either be due to how the mineral grew during the deposition process or it could also indicate that mineral reabsorption occurs inside the deposits. In the HO deposits that were bulging out of the tendon, some of the unmineralized areas contained spherical structures reassembling the adipose tissue on the outside of the tendons (Fig. 2D). This could indicate that some adipose tissue has been encapsulated within the HO deposits during mineral deposition.

This study also investigated the feasibility of using needling of intact rat tendons to study the relationship between inducing microtrauma and HO. The data collected in this study indicate that the volume fraction of HO deposits in needled tendons might be higher than in the control group, which is also supported by the HO deposits being close to the microdamage. One further interesting observation for needled tendons was the merging of HO deposits which seems to progress through mineral accretion along some of the unmineralized fibers in between the deposits. The merging was exemplified as three distinct deposits growing towards each other including mineralized regions that could be part of either one of the deposits (Fig. 4). Thus, over time smaller HO deposits may merge to form bigger deposits. While small HO deposits in tendons may have little effect on its mechanical properties, large mineralized regions could affect the mechanical performance of the tendon substantially (O’Brien et al., 2013).

In previous studies, laboratory based μCT was used to establish the location of HO deposition in rodent Achilles tendons and the overall geometry of the bigger deposits (O’Brien et al., 2012, O’Brien et al., 2013, Lin et al., 2018, Huber et al., 2020). However, the limited resolution did not enable them to provide details about the deposits internal structure. Additionally, thanks to the phase contrast enhancement in SR-PhC-μCT, we were able to acquire information about the microstructure of the tendon soft tissue surrounding and within the deposits. We could show the transition between unmineralized collagen fibers to mineralized tissue, and thereby capturing the very early stages of mineral formation and investigate the relation between deposition and cells. None of these can be well resolved in conventional μCT.

Species specific differences exist for HO deposition in tendons (O’Brien et al., 2012). For instance, mice develop tendon mineralization with ageing while rats appear to develop HO from a young age. Consequently, in this study all intact rat tendons contained HO deposits, whereas in previous studies, mice tendons from the control group did not contain any big HO deposits (O’Brien et al., 2012, O’Brien et al., 2013). The presence of pre-existing deposits makes it harder to identify the effect of the needling in intact rat tendons. In the future, further investigation using younger rats could help understanding when HO deposition starts in intact rat Achilles tendons. Using younger rats, and consequently having a control group totally lacking HO deposits, could possibly improve our understanding of the relation between microdamage and HO formation. In previous studies, HO formation was reported in needled mouse tendons after 20 weeks (O’Brien et al., 2012, O’Brien et al., 2013). In our study the rats were sacrificed 4 weeks after needling in the attempt to capture the very early stages of HO deposition. One sample had indeed HO deposited in the injured area, but the remaining samples had not, and this indicate that HO is primarily occurring after the first month. Consequently, it could be beneficial to extend the time between needling and the end point of the experiment to assess a definitive correlation between the needling and new HO deposition. Additionally, it should also be noted that the previously discussed study on mouse Achilles tendons, found an increased bilateral HO occurrence after needling together with changes in mechanical properties (O’Brien et al., 2013). In the future, it would be interesting to investigate if also rat contralateral Achilles tendons present an increase in HO and a different mechanical response compared to controls.

Using cutting-edge synchrotron-based imaging techniques implies restricted experimental time, which limits the number of samples that can be studied. Since the micro-injuring of tendons by needling was an exploratory study, we restrained the sample number to 3 per group, and to one time point. Furthermore, only female rats were considered as they grow slower than male rats. Therefore, we do not attempt to study sex specific differences. As the results show great potential of using phase contrast enhanced synchrotron tomography to detect and track induced microdamage as well as HO depositions, future studies should involve more animals to investigate the dynamic nature of mineral deposition induced by needling over time and possible gender specific differences.

Regardless of the species-specific differences discussed above, similarities between mouse and rat models can be noted. In both species HO deposits had a similar overall shape and were identified close to the calcaneus bone. Furthermore, in both species some of the HO deposits were observed very close to the tendon surface, almost bulging out from it. The exact mechanism guiding HO is not clear. However, HO deposits in Achilles tendons are not unique to rats and mice but are also found in humans, most often in healing tendons on the anterior side (Magnusson et al., 2020). In general, HO deposits seem to be more commonly observed close to the calcaneus, often near the surface, and in some cases protruding out from the stump. The fact that the HO deposits are most often found in the distal part of the tendon could possibly be due to the vicinity of the calcaneus where bone is physiologically present. Previous literature has also suggested that tissue vascularization precedes HO (Dilling et al., 2010, Hwang et al., 2019). Consequently, one of the reasons why HO deposits are mostly found in superficial locations could be due to the higher blood supply in the vicinity of the tendon’s surface. Furthermore, studies have shown that the anterior superficial part of the Achilles tendon elongates and strains more than the posterior part in both humans and rats (Finni et al., 2018, Bogaerts et al., 2017). Consequently, this region could be more prone to microdamage which, in turn, could trigger HO deposition. Further investigation of the complex relation between mechanical stimulation, local strain, microdamage and pathological mineralization could be of great interest to fully understand the complex factors leading to deposition of HO.

Although synchrotron X-ray tomography is currently not a diagnostic tool and can only be performed on a limited number of samples, we believe that the presented results can pave the way for future studies using high-resolution synchrotron X-ray tomography to understand the mechanisms by which HO occurs and how interventions may alter the progression of HO dynamically.

## 5 Conclusions

HO is associated with tendinopathy and may result in discomfort and impaired mechanical properties. Our results show that HO deposits are present in intact rat Achilles tendons, and that they are characterized by a unique structure in which some fibers in the tendon soft collagen matrix have mineralized. Moreover, HO deposition seems to initiate in the intercellular space and propagate along fibers in between cells. We also show that needling of intact tendons in combination with phase contrast enhanced synchrotron X-ray tomography is a promising approach to determine the relation between microdamage at the collagen fiber level and HO.

## CRediT authorship contribution statement

**Maria Pierantoni:** Conceptualization, Investigation, Methodology, Validation, Writing – original draft. **Malin Hammerman:** Data curation, Investigation, Validation, Writing - review & editing. **Isabella Silva Barreto:** Data curation, Validation, Writing – review & editing. **Linnea Andersson:** Data curation. **Vladimir Novak:** Data curation, Methodology, Resources, Writing – review & editing. **Hanna Isaksson:** Conceptualization, Data curation, Investigation, Funding acquisition, Supervision, Writing – review & editing. **Pernilla Eliasson:** Conceptualization, Data curation, Investigation, Funding acquisition, Writing – review & editing.

## Declaration of Competing Interest

The authors declare that they have no known competing financial interests or personal relationships that could have appeared to influence the work reported in this paper.

## Data Availability

Data will be made available on request.
